# Phospholipase C activity increases in cerebrospinal fluid from migraineurs in proportion to the number of comorbid conditions: a case–control study

**DOI:** 10.1186/1129-2377-14-60

**Published:** 2013-07-04

**Authors:** Alfred N Fonteh, Janice M Pogoda, Rainbow Chung, Robert P Cowan, Michael G Harrington

**Affiliations:** 1 Molecular Neurology Program, Huntington Medical Research Institutes, 99 N El Molino Ave, Pasadena, CA 91101, USA; 2 Statology, 158 W Cooke Rd, Columbus, OH 43214, USA; 3 Department of Neurology, Stanford University School of Medicine, Stanford, CA 94305, USA; 4 University of California, Irvine School of Medicine, Irvine, CA 92697, USA

**Keywords:** CSF, PLC, Depression, Irritable bowel syndrome, Painful bladder

## Abstract

**Background:**

Migraineurs are more often afflicted by comorbid conditions than those without primary headache disorders, though the linking pathophysiological mechanism(s) is not known. We previously reported that phosphatidylcholine-specific phospholipase C (PC-PLC) activity in cerebrospinal fluid (CSF) increased during migraine compared to the same individual’s well state. Here, we examined whether PC-PLC activity from a larger group of well-state migraineurs is related to the number of their migraine comorbidities.

**Methods:**

In a case–control study, migraineurs were diagnosed using International Headache Society criteria, and controls had no primary headache disorder or family history of migraine. Medication use, migraine frequency, and physician-diagnosed comorbidities were recorded for all participants. Lumbar CSF was collected between the hours of 1 and 5 pm, examined immediately for cells and total protein, and stored at −80°C. PC-PLC activity in thawed CSF was measured using a fluorometric enzyme assay. Multivariable logistic regression was used to evaluate age, gender, medication use, migraine frequency, personality scores, and comorbidities as potential predictors of PC-PLC activity in CSF.

**Results:**

A total of 18 migraineurs-without-aura and 17 controls participated. In a multivariable analysis, only the number of comorbidities was related to PC-PLC activity in CSF, and only in migraineurs [parameter estimate (standard error) = 1.77, p = 0.009].

**Conclusion:**

PC-PLC activity in CSF increases with increasing number of comorbidities in migraine-without-aura. These data support involvement of a common lipid signaling pathway in migraine and in the comorbid conditions.

## Background

Migraineurs are prone to a large variety of comorbidities [1]. Depression, the most common comorbid condition, and migraine are among the highest ranked causes of global years lived with disability [2]. Other comorbidities include cardiovascular, respiratory, gastrointestinal, neurological, and psychiatric conditions as well as osteoarthritis and allergies [3-6]. Recent reports associated restless legs and sensorineural hearing loss with migraine [7,8]. The molecular associations that are common to these disorders are not known, but include shared genetic risk factors [6,9], regulation of brain cations [10], or common receptor signaling events that activate pain [11], inflammatory [12], or oxidative [13] pathways. The same neurotransmitter acting at different sites may also underlie varied disorders; for example, serotonin and endocannabinoids are implicated in both depression and migraine [14,15].

To consider common biochemical pathways, migraine and many of its comorbidities have been found to share alterations in serotonin [16], noradrenaline [17,18], estrogen [19], cannabinoids [20,21], and glutamate [22,23]. Medications that modulate the G-protein coupled receptors (GPCRs) for these ligands may alleviate symptoms of migraine and comorbidities [24,25]. Since the signaling pathways for these ligand-bound GPCRs involve PC-PLC activation [26-28], we hypothesize that migraine and its comorbidities share a common pathway of receptor-mediated, lipid second-messengers that are regulated by PLC activation. Migraine model studies further implicate PLC pathway involvement: the migraine trigger pituitary adenylate cyclase activating peptide-38 induced mast cell degranulation in the rat dura mater, mediated by the PLC pathway [29]; and a homolog of the familial hemiplegic migraine candidate gene 2 in *C. elegans* (EAT-6) interacts with a homolog (EGL-8) of PLC [30]. Similarly, newly identified peptides such as orexin and ghrelin, which are known to regulate sleep-wakefulness, mediate their excitatory effects on the pedunculopontine tegmental nucleus by PLC activation [31].

We tested the hypothesis that migraine and its comorbidities share a common receptor-mediated pathway. We compared migraineurs and non-headache suffering controls on the relationship between PC-PLC activity in CSF and the number of different comorbidities experienced. Our hypothesis of PC-PLC dysregulation predicts that a person with a more extensive number of comorbid conditions would have greater deviation of PC-PLC activity within the nervous system, and this would be reflected in the CSF.

## Methods

This case–control study, conducted between 2004 and 2011, was designed to measure PC-PLC activity in lumbar CSF from migraineurs (cases) and non-headache suffering controls and to associate it with the number of physician diagnosed conditions known to be comorbid with migraine. The Huntington Hospital Institutional Review Board for Human Research approved the study protocol and consent forms which all participants signed.

### Study participants

Consecutive participants between 18 and 75 years of age were recruited from our research clinic and from the Pasadena area through local advertising and research presentations. All controls and migraineur participants were recruited prospectively for this study to our research institute. After informed consent was given, we assessed them using structured interviews and by clinical examination. Our goal was to recruit as many participants as possible over the time period. We excluded participants with any contraindication for lumbar puncture (e.g., warfarin) or with any additional, uncontrolled diagnosis identified from the assessments. They returned for CSF collection within 2 weeks of completing clinical assessment.

Headache diagnosis was determined by consensus after clinical assessment by one of two migraine diagnosticians (RPC or MGH). To minimize heterogeneity, migraineurs were included as cases only when the primary headache disorder was migraine-without-aura, as defined by the criteria of the International Classification for Headache Disorders, 2nd edition (ICHD-2 code 1.1). Additional case inclusion criteria were: at least 1 migraine per month and less than 15 headache days per month; availability of data on duration of migraine and attack frequency for the preceding year; all medications recorded for 90 days before and throughout the study; no change in prophylactic medications during the 90 days before study; and no rescue medication taken within 48 hours of sample collection. Migraine frequency for the preceding 12 months was recorded from that estimated by each participant. Migraine intensity, only used for diagnosis, was recorded from a subjective 0 – 10 scale, without treatment when available, for their range of migraine at typical, mild, and most severe episodes.

Inclusion criteria for healthy controls were: absence of primary headache disorder after the same assessments as migraineurs; no family history of migraine in first-degree relatives; and no change in prophylactic medications for 90 days before and throughout the study.

### Data collection

#### Comorbidities

Participants were required to complete an extensive structured interview for medical and neurological disorders and medical and neurological examinations to document any other conditions, including comorbid diagnosis. Body mass index (BMI) was calculated from height and weight ascertained at study enrollment. The Structured Clinical Interview for DSM-IV Axis I Disorders, Research Version/Non-patient Edition, was administered to participants to evaluate major psychiatric diagnosis (SCID-I) and the Axis II Disorders (SCID-II) interview was administered to diagnose personality disorders. We assessed personality in more detail using the 1989 revision of the Minnesota Multiphase Personality Inventory (MMPI-2). We paid particular attention to the first three clinical scales dealing with concern for bodily symptoms that have been reported to differ in migraine [32]: Scale 1 (Hypochondriasis), Scale 2 (Depression), and Scale 3 (Hysteria). To determine subjective symptoms and severity of depression, participants rated themselves using the Beck Depression Inventory, 2^nd^ edition (BDI-2) [33]. Prior comorbidities were based on diagnosis by a specialist physician, independent of the migraine/control diagnosis by the research clinician: depression (psychiatrist), anxiety (psychiatrist), epilepsy (neurologist), allergy (allergist or internist), asthma (internist), eczema (dermatologist), irritable bowel syndrome (gastroenterologist or internist), fibromyalgia (rheumatologist or internist), patent foramen ovale (cardiologist), mitral valve prolapse (cardiologist), hypertension (internist or cardiologist), osteoarthritis (rheumatologist or internist), and painful bladder syndrome (urologist).

Having a single investigator record assessment of comorbidities throughout the study, and using a structured interview list to obtain responses to direct questions of physician-diagnosis for each known comorbid condition controlled potential bias in the ascertainment of comorbid diagnoses for all participants. It was not practical for the investigator to be blind to case–control status, as this status was usually described during the assessment.

#### CSF

Migraineurs had no headache (0 of 0–10 scale) for > 24 hours at the time of CSF collection. All CSF samples were collected between 1:00 and 6:00 pm to minimize chronobiological variation. CSF was obtained by lumbar puncture using a 22-gauge Quincke-type needle, centrifuged (3000 g for 3 min) for cell count, and the supernatant fluid was stored at −80° C until thawed for PC-PLC or total protein assay. The same fraction of CSF from three sequential fractions taken from each participant was analyzed in this study to reduce variation from the neuraxis gradient.

#### Laboratory

Cells were counted in a hemocytometer with trypan blue exclusion. Total protein concentrations were determined with the fluorescent Quant-iT™ protein assay kit (Invitrogen/Molecular Probes, Eugene, OR) with bovine serum albumin (0–500 ng/ml) as a standard for quantification. Fluorescence (excitation at 470 nm and emission at 570 nm) was measured using a Gemini XPS Dual-Scanning Microplate Spectrofluorometer and data analyzed using SoftMax® Pro software (Molecular Devices, Sunnyvale, CA).

#### PC-PLC assay

Amplex® Red PC-PLC Assay Kit (A12218, Invitrogen/Molecular Probes) was used to measure enzyme activity in CSF. PC-PLC from *Bacillus cereus* was used as a positive control to estimate PLC protein content of CSF. CSF was diluted in sample buffer (50 mM Tris–HCl (pH 7.4), 0.14 M NaCl, 10 mM dimethylglutarate, 2 mM CaCl_2_) to ~0.1 mg/ml, and placed in 96 well plates in triplicates. Adding 100 μl Amplex Red working solution to 100 μL of the diluted CSF samples started PLC activity. The plate was incubated at 37°C with monitoring of fluorescence using excitation at 545 nm and emission detection at 590 nm every 5 min for 90 min. An increase in fluorescence due to PLC-induced release of phosphocholine was recorded and specific activity calculated as Relative Fluorescence Unit (RFU) per microgram protein per min (RFU/mg/min).

Potential bias in the collection of PC-PLC activity levels was controlled by randomly assigning samples from migraineurs and controls to plates by a single investigator and by running and analyzing samples in batches by a separate investigator who was blinded to case–control status.

### Statistical analysis

Complete data were obtained for all participants. Group comparisons were done using Fisher’s exact tests for categorical variables and Wilcoxon rank sum or Student’s t tests, as appropriate, for continuous variables. All analyses were univariable unless otherwise stated. Multivariable analyses, including computation of least squares (LS) means and standard errors (SEs) were done using general linear models [34]. Analyses were done using SAS v9.2 (SAS Institute, Inc., Cary, NC). All tests were two-sided with 0.05 significance levels.

## Results

### Clinical findings

A total of 35 study participants (17 controls and 18 migraineurs-without-aura) met study inclusion criteria and consented to the study. All participants completed the study without adverse effects from the lumbar puncture. Table 1 describes the clinical characteristics, CSF cell counts and total protein. Controls were older (p = 0.01) and had a greater percentage of males (p = 0.15) than migraineurs. As expected, controls took fewer prescription medications (p = 0.0001) and, in contrast with some reports [35], had higher BMI (p = 0.03) than migraineurs. MMPI Scales 1–3 and BDI-2 scores were significantly higher in migraineurs than in controls. None of the controls or migraineurs had episodes of Axis I depression within 3 months before assessment. CSF cell counts and total proteins were within normal ranges in both controls and migraineurs.

**Table 1:** Clinical characteristics, cerebrospinal fluid (CSF) cell counts and total protein by diagnostic group

	**n**	**Controls**	**n**	**Migraineurs**	**P-value**
Men, n (%)		7 (41)		3 (17)	0.15
Women, n (%)		10 (59)		15 (83)	
Age (years), mean (SD)	17	62.1 (18.6)	18	46.9 (15.7)	0.01
Body mass index (kg/m^2^), mean (SD)	17	26.4 (3.4)	16	23.4 (4.1)	0.03
# Comorbidities, mean (SD)	17	1.6 (1.2)	18	1.9 (1.2)	0.47
# Medicines, mean (SD)	17	0.35 (0.49)	18	5.3 (4.4)	0.0001
MMPI Scale 1, mean (SD)	11	50.3 (7.8)	15	59.3 (9.0)	0.01
MMPI Scale 2, mean (SD)	11	47.3 (6.1)	16	56.0 (10.3)	0.01
MMPI Scale 3, mean (SD)	11	49.2 (5.9)	16	57.6 (14.0)	0.04
BDI-2, mean (SD)	14	3.9 (3.6)	17	8.5 (5.2)	0.009
CSF cell count	17	< 5 per mL	18	< 5 per mL	
CSF total protein, g/L (SD)	17	0.36 (0.11)	18	0.36 (0.12)	0.78

*MMPI* Minnesota Multiphasic Personality Inventory, *BDI-2* Beck Depression Inventory-2.

Frequencies of individual comorbidities are shown in Table 2. Total number of comorbidities per participant group was higher in migraineurs than controls, though not significantly. Depression was twice as common in the migraineurs.

**Table 2:** Number of comorbidities, diagnosed by a specialist, by diagnostic group

**Comorbidities**	**Controls (n = 17)**	**Migraineurs (n = 18)**
Depression	5 (29%)	10 (56%)
Anxiety	0	1 (6%)
Epilepsy	1 (6%)	0
Hypertension	4 (24%)	3 (17%)
Irritable bowel syndrome	2 (12%)	3 (17%)
Mitral valve prolapse	1 (6%)	1 (6%)
Patent foramen ovale	1 (6%)	2 (11%)
Osteoarthritis	5 (29%)	6 (33%)
Painful bladder syndrome	1 (6%)	4 (22%)
Fibromyalgia	1 (6%)	1 (6%)
Asthma/allergy	3 (18%)	2 (11%)
Eczema	0	1 (6%)
**Total**	**24**	**34**

### CSF PC-PLC activity

Mean PC-PLC activity levels were similar in migraineurs [mean (SD) = 9.79 (3.42) RFU/mg/min] and controls [mean 9.29 (1.97) RFU/mg/min]. In a multivariable model with age and sex as independent variables, among controls age was inversely related to PC-PLC (parameter estimate (SE) = −0.045 (0.022), p = 0.054) and males had lower PC-PLC than females [LS mean (SE) = 7.9 (0.60) males, 10.2 (0.50) females; p = 0.01); among migraineurs, age was not related to PC-PLC activity (p = 0.99) and the difference in LS means by sex was similar to that in controls [LS mean (SE) = 7.6 (2.10) males, 10.2 (0.91) females], though non-significant (p = 0.28). Thus, the lack of difference between migraineurs and controls in PC-PLC was the same regardless of sex.

In a multivariable analysis of migraineurs that considered sex, number of medications used, migraine frequency (days) in the past year, psychiatric measures, and number of comorbidities as potential predictors of PC-PLC activity, only number of comorbidities was independently significantly related to PC-PLC (parameter estimate (SE) = 1.77 (0.58), p = 0.009; Table 3). Only 2 migraineurs had no comorbidities. With number of comorbidities dichotomized as 0–1 and > 1, PC-PLC did not differ by sex among migraineurs in the 0–1 comorbidity group, and there are no men in the > 1 comorbidity group.

**Table 3:** CSF PC-PLC activity levels (RFU/mg/min) in migraineurs by number of comorbidities

**Number of comorbidities**	**Number of migraineurs**	**PC-PLC levels mean (SD)**
0 or 1	7	7.5 (1.5)
2	6	9.9 (3.8)
> 2	5	12.9 (2.7)

In a multivariable analysis of controls that considered the same potential predictors as above except migraine frequency, only age and sex were significant independent predictors.

## Discussion

We observed that PC-PLC activity was positively correlated with the number of comorbid conditions in migraineurs but not in controls, supporting our hypothesis that PC-PLC activity is part of a common pathway in the comorbid conditions of migraineurs. This first description of a shared involvement of CSF PC-PLC in these comorbid conditions is significant when it is considered that many helpful medications modulate the biochemical pathways that also share involvement in these conditions, such as serotonin [16] or noradrenaline [17,18]. This inference raises the possibility that modulators of PC-PLC may help lessen migraine, as well as other comorbid conditions.

We also observed greater PC-PLC activity in CSF in females in both migraineurs and controls and, among controls only, in younger participants. Given the higher PC-PLC activity in migraineurs compared to controls, the sex difference we observed is consistent with the higher incidence of migraine in the female population.

In considering how PLC activity may relate to migraine and its comorbidities, many of these distinct conditions share a multifaceted pathophysiology, with alterations in molecules as diverse as serotonin, adrenaline, estrogen, cannabinoids, and glutamate, and include the activation of several GPCRs, protein kinase mediated signaling, and early gene activation (c-fos) [36,37]. PC-PLC plays a role in this process by generating the protein kinase activator, diacylglycerol, and affecting Ca^2+^ influx into cells [38,39]. We hypothesize that varying PC-PLC levels, as we observed in our study, reflect fluctuations from their modulation of many G protein coupled receptors, and that any changes of PC-PLC activity in the brain are reflected in CSF. Once altered, PC-PLC can have diverse effects. For example, signaling pathways induced by PC-PLC can form inflammatory or pain molecules; also, PC-PLC can hydrolyze PC, a major component of membrane lipids, altering membrane properties and the functions of ion channels imbedded in the lipid bilayer [40]. Our data suggest that the diverse pain, inflammatory, and behavioral features common to migraine and its comorbidities may arise at least partially from altered PC-PLC activity. Many different CSF PLC isoforms are yet to be defined, but their roles in vascular endothelial dysfunction and inflammation can be explored for therapy [41]. Thus, future studies of isoform-specific PLC modulation may lead to treatments for migraine and its comorbid conditions.

The invasiveness of collecting lumbar CSF in a prospective study led to a relatively small study population (n = 35) and interpreting results from a multivariable analysis can lead to not only bias but to under- or over-estimated variance; this, combined with the clinical differences we observed between controls and migraineurs (such as differences in medication use), necessitates cautious interpretation of our findings. For example, the small numbers for each individual comorbidity (Table 2) means we were unable to analyze the CSF PC-PLC activity levels for any individual comorbidity or to fully explore possible confounders and modifiers of the association between PC-PLC activity and comorbidities. We used physician-diagnosed comorbidities which probably underrepresent some diagnoses; however, this should have occurred randomly in both migraineurs and controls and without regard for PC-PLC activity levels, likely leading to attenuated estimates of the relationships between migraine, comorbidities, and PC-PLC activity. Furthermore, using physician-diagnosed comorbidities minimized the potential bias associated with unblinded participant interviews to ascertain medical histories.

## Conclusions

We report that the PC-PLC activity in CSF from participants with migraine-without-aura, but not for non-headache suffering controls, increases in proportion to the number of migraine comorbidities. These findings offer the potential insight of PC-PLC signaling as a common pathway in migraine and its comorbidities.

## Abbreviations

PC: Phosphatidylcholine; PLC: Phospholipase C; CSF: Cerebrospinal fluid; GPCR: G-protein coupled receptor; BMI: Body mass index; SCID-1 & 2: Structured Clinical Interview for DSM-IV Axis I Disorders, Research Version/Non-patient Edition, Axes 1 & 2; MMPI-2: Minnesota Multiphasic Personality Inventory; BDI-2: Beck depression inventory, 2^nd^ edition; RFU: Relative fluorescence unit; LS: Least squares; SE: Standard errors.

## Competing interests

All authors declare that they do not have any competing interest.

## Authors’ contributions

ANF designed the PC-PLC assay, participated in the overall design of the study, and drafted the manuscript. JMP participated in the design of the study, executed the statistical analyses, and drafted the manuscript. RC carried out the PC-PLC assays. RPC participated in the design of the study and recruited study participants. MGH conceived of the study, participated in its design and coordination, recruited the study participants, and drafted the manuscript. All authors read and approved the final manuscript.
